# Enhancing Self-Esteem, Well-Being, and Relaxation in the Elderly through Nature-Based Interventions

**DOI:** 10.3390/ijerph21070952

**Published:** 2024-07-20

**Authors:** Anna Heród, Bożena Szewczyk-Taranek, Bożena Pawłowska

**Affiliations:** Department of Ornamental Plants and Garden Art, University of Agriculture in Krakow, 29-Listopada 54, 31-425 Kraków, Poland; bozena.szewczyk-taranek@urk.edu.pl (B.S.-T.); bozena.pawlowska@urk.edu.pl (B.P.)

**Keywords:** horticultural therapy, older adults, long-term care facilities, nursery, garden therapy

## Abstract

As the populations of many countries are aging, institutions providing support for the elderly increasingly often use nature-based interventions (NBIs) as part of their therapeutic activities. This study aimed to show the impact of 8-week active and passive NBI programs on self-esteem, well-being, and relaxation in the elderly. The NBI programs were conducted in two groups of elderly citizens of Poland: independent senior students and seniors requiring 24 h care. The Rosenberg’s Self-Esteem Scale and the World Health Organization Well-Being Index were used to assess self-esteem and well-being before and after the programs. To assess relaxation, pre–post session measurements of pulse rate (PR) and blood oxygen saturation (SpO_2_) were used. Our study showed an improvement in the self-esteem, well-being, and relaxation of the participants of both active and passive NBI programs. In all study groups, self-esteem and well-being improved after the programs, regardless of the type of intervention, and after each NBI session, the elderly showed a decrease in PR and an increase in SpO_2_, which indicated a deeper state of relaxation. Our research showed a greater improvement in the tested parameters in the seniors participating in the passive programs in a garden. This confirms a key role of green spaces in residential areas for the elderly and in nursing homes.

## 1. Introduction

Life expectancy is steadily growing, which affects the population age structure in many countries [1]. Older age brings about progressive changes in the tissues and organs that lead to a decline in functioning and the development of many diseases [2]. A topic attracting the attention of researchers is healthy aging, the definition of which encompasses not only being free of chronic diseases but also covers domains such as survival to a specific age, autonomy in activities of daily living, well-being, good quality of life, high social participation, only mild cognitive or functional impairment, and little or no disability [3].

The boost in life expectancy is accompanied by a global increase in the provision of institutional services aimed at providing support to the elderly at various stages of their life. Therefore, numerous institutions take actions aimed at maintaining the physical and mental health of the elderly by pursuing their passions, continuing education, having an active lifestyle, and later providing necessary care [1]. European health and social care systems vary depending on the national social policy but are most often based on three pillars: community- and family-based care, primary and social care, and long-term and hospital care [4]. In Poland, social policy aimed at the elderly encompasses various forms of community, institutional, and semi-institutional assistance. Older people who live independently within their community can attend classes organized by seniors clubs or seniors activity centers, as well as widely popular Universities of the Third Age (U3As). The first U3A was established by Professor Pierre Vellasa in 1973 at the Toulouse University of Social Sciences in France. Its success resulted in the rapid development of U3A in other countries. By 1975, large academic centers in Belgium, Poland, Switzerland, and Italy had their own U3A. The activities offered by U3A are aimed at the education and intellectual development of the elderly, pursuing their passions and their social inclusion. Another group of older people are the elderly that require 24 h care. Depending on their condition, the elderly are living in long-term care facilities (LTCFs) that provide care, living, and educational services to people who need 24 h care due to age, disease, or disability [5,6].

Institutions intended for older people offer integrative and therapeutic activities that aim to improve well-being and good mental condition and are tailored to the abilities and interests of the participants. These are the three most often used forms of therapy. These include art therapy, that is therapy through art, encompassing, for example, plastic arts therapy; ergotherapy, that is therapy through work and recreation, for example, gardening; and social therapy, that is therapy through social interactions [7]. The activation and therapeutic classes for the elderly increasingly offer activities involving therapeutic horticulture that combine different forms of stimulation. Therapeutic horticulture is the process through which participants enhance their well-being by involvement in gardening and plant-related activities [8]. Therapeutic horticulture may be active or passive, and the classes may take place indoors or outdoors. The active classes focused on therapeutic horticulture most often include cultivation techniques, floristry, and artistic creations, while the passive classes offer plant viewing and touching, listening to and smelling the landscape, breathing exercises, and mindfulness. The class scenarios are always prepared for specific groups, and they take into account the abilities, interests, and previously defined therapeutic goals of individual participants [9].

This article aims to contribute to the body of literature by showing the impact of horticultural therapy classes on improving self-esteem, well-being, and the state of relaxation in older people. Self-developed nature-based intervention (NBI) programs were conducted in two groups of seniors from Poland: (1) independent U3A students and (2) seniors requiring 24 h care. The seniors participated in either active or passive NBI programs. The Rosenberg’s Self-Esteem Scale and the World Health Organization Well-Being Index were used to assess the psychological parameters of self-esteem and well-being. The tests were conducted before and after each program consisting of eight sessions. The impact of NBI programs on the state of relaxation was assessed based on physiological measurements of pulse rate (PR) and blood oxygen saturation (SpO_2_) performed before and after each 1.5 h session.

## 2. Materials and Methods

### 2.1. Participants

Nature-based intervention programs involved senior students of a U3A, who are healthy or subhealthy people living independently and unassisted in their communities and senior residents of an LTCF, who are people living in facilities providing 24 h care.

The NBI programs were attended by people aged at least 60 years being either U3A students or senior residents of an LTCF for at least 3 months, without any problems with verbal communication or diagnosed mental illnesses. There were 48 participants, aged 61 to 95 years (mean age 75.6 ± 8.8 years). Both groups consisted of an equal number of 24 participants. Most of the participants were women, who accounted for 91.7% of the senior students of the U3A and 87.5% of the senior residents of the LTCF. The mean age was 70.8 ± 4.6 for the U3A senior students and 80.4 ± 9.5 for the LTCF senior residents (Table 1).

### 2.2. Procedure of Nature-Based Interventions Included in the Programs

The NBI programs were designed to support the mental, social, and cognitive aspects of well-being, and the activities were selected based on surveys on gardening interests in older people [10].

**Figure 1:**
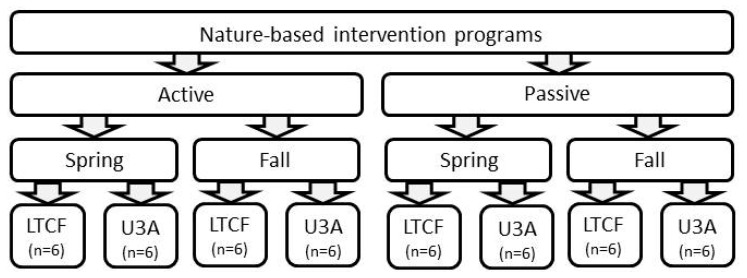
Design of the nature-based intervention programs for the residents of the long-term care facility (LTCF) and students of the University of the Third Age (U3A) during the spring and fall 2023.

The NBI programs included 8 active or passive sessions. They were conducted twice in 2023, in the spring and fall. The programs were implemented separately for the U3A students and the LTCF residents. A total of eight groups participated in the NBI programs, each consisting of 6 participants (Figure 1). Each person could take part in the NBI program (8 sessions) only once.

Before the start of each NBI program, the participants’ self-esteem and well-being were measured using the Rosenberg’s Section “Self-Esteem” Scale and the World Health Organization Section “Well-Being” Index. Each session lasted approximately 1.5 h and began with an introduction during which the participants had their pulse rate and oxygen saturation measured. After that, the session scenario was pursued, which for the active sessions included the explanation and execution of planned activities. During the passive sessions, breathing exercises were performed to oxygenate the body and achieve a state of relaxation, and time was spent outdoors where the participants performed sensory exercises. All sessions ended with a summary, during which the seniors could share their reflections, and another measurement of pulse rate and oxygen saturation was taken. The sessions were conducted by a therapist supported by a researcher and an assistant. The sessions for the U3A students were held at the university campus et al. 29 Listopada in Kraków, and those for the LTCF residents took place at the premises of the LTCF at Kluzeka 6 in Kraków.

The active sessions were divided into three types: indoor gardening (sessions 1–2), plant-related art (sessions 3–5), and floristry (sessions 6–8; Table 2). Indoor gardening was selected for the sense of accomplishment provided by successful plant cultivation and the possibility to reflect on the meaning of life by watching the plants grow. The sessions were held indoors due to the limited space available at the U3A and LTCF. Easy-to-grow plant species were selected for sowing, such as tomato (*Solanum lycopersicum*), cucumber (*Cucumis sativus*), radish (*Raphanus sativus* var. *sativus*), endive (*Cichorium endivia*), rocket salad (*Eruca vesicaria * ssp. *sativa*), sunflower (*Helianthus annuus*), and marigold (*Tagetes erecta*). For propagation, we chose popular pot plants with sensory properties, such as dayflower (*Tradescantia* sp.), pilea (*Pilea* sp.), philodendron (*Philodendron* sp.), snake plant (*Sansevieria* sp.), African violet (*Saintpaulia* sp.), or spider plant (*Chlorophytum* sp.). During the session, the participants were provided with information on the cultivation and care of individual plant species, so that they could properly take care of them after the program. Plant-related art focused mainly on training manual skills, stimulating participants’ creativity, and increasing social interactions. During the sessions, the participants performed dried flower decorations (paintings), decoupage flower pots, and paper succulents that were then “planted” in the pots. Over the sessions, the participants were encouraged to exchange opinions on their works and help each other. During the floristic sessions, the rooms where the programs took place were filled with large amounts of flowers. This allowed everyone to admire the beauty of nature and reflect on memories from their youth. The species used during the sessions were those at the peak of their vegetation in a given season. In the spring, they were, for example, peony (*Paeonia* sp.), coral bells (*Heuchera* sp.), common columbine (*Aquilegia vulgaris*), cornel (*Cornus* sp.), geranium (*Geranium* sp.), sage (*Salvia* sp.), campion (*Lychnis* sp.), Lady’s mantle (*Alchemilla vulgaris*), or lavender (*Lavandula angustifolia*), and in the fall, rose (*Rosa* sp.), dahlia (*Dahlia* sp.), gladioli (*Gladiolus* sp.), ninebark (*Physocarpus opulifolius*), false spirea (*Astilbe thunbergii*), ox-eye daisy (*Leucanthemum vulgare)*, aster (*Aster* sp.), or purple coneflower (*Echinacea purpurea*). When selecting the plants, we took into account the various colors, textures, and scents to ensure a variety of sensory experiences. We also chose plants that are not toxic and with no allergic potential with skin contact. If the juices of the plant have an allergic potential e.g., philodendron (*Philodendron* sp.), participants were informed in advance, and the plant was used with caution. The course of an example session of the active NBI program is presented in Table 3.

In the passive NBI programs, the sessions focused mainly on sensory stimulation and mindfulness practice. They took place outdoors, in green areas belonging to the U3A and LTCF. They were divided into two types: sensory walks (sessions 1–6) and mindfulness walks (sessions 7–8; Table 2). During the sensory walks, the participants performed exercises engaging individual senses. These included noticing the colors and patterns of nature, concentrating on the soundscape, recognizing the smells of the surroundings, learning about the natural textures of the surroundings, and recalling favorite flavors from their childhood garden. Mindfulness walks were focused on promoting a positive mindset. Activities in this part included breathing exercises aimed at achieving a state of relaxation, including the 3-3-6 breathing technique, which involves focusing on breathing by taking a 3 s deep breath through the nose, holding the breath for 3 s, and exhaling through the mouth for 6 s. Additionally, the participants listed things for which they were grateful, things that made them happy, and beautiful things in their surroundings. The course of an example session of the passive NBI program is presented in Table 4.

After the completion of each NBI program, the participants’ self-esteem and well-being were measured again using the Rosenberg’s Section “Self-Esteem” Scale and the World Health Organization Section “Well-Being” Index.

### 2.3. Outcome Measurements

The NBI programs assessed changes in the psychological parameters self-esteem and well-being and the physiological parameters pulse rate (PR) and blood oxygen saturation (SpO_2_). Self-esteem and well-being were measured before and after each NBI program consisting of 8 sessions (a total of 192 measurements), while PR and SpO_2_ were measured before and after each 1.5 h session (a total of 1536 measurements).

#### 2.3.1. Psychological Parameters

The psychological parameters were assessed by the researcher and the assistant, who read questions to the participants and marked their answers on a paper version of the form.

##### Self-Esteem

Self-esteem was evaluated using the Polish version of the Rosenberg’s Self-Esteem Scale (SES) [11]. The SES consists of 10 questions measuring overall self-esteem, based on both positive and negative feelings about one’s self. Answers are given as per a 4-point Likert scale. The possible score ranges from 10 to 40 points, and the higher the score, the higher the self-esteem. The mean score for 53 countries is 30.85, and for Poland, it is 29.49 [11,12].

##### Well-Being

Well-being was evaluated using the Polish version of the World Health Organization Well-Being Index (WHO-5). The WHO-5 includes five statements on the subjective quality of life based on positive mood, vitality, and general interest. Respondents rate the statements according to a 6-point scale. A result below 12.5 points indicates poor well-being and is a basis for a diagnosis of depression [13]. An average score for European countries is around 17.5 points [14]. In order to monitor changes in well-being, the percentage score is used [15].

#### 2.3.2. Physiological Parameters

##### Pulse Rate and Blood Oxygen Saturation

Changes in PR and blood SpO_2_ before and after each session were assessed with a TM-PX30 pulse oximeter from TECH-MED, Poland. The measurements were carried out as recommended by the manufacturer. All measurements were performed only once. Normal resting PR in adults ranges from 60 to 100 bpm. Its increase may be due to greater physical activity, stress, or a sense of danger, while a lower PR indicates a state of rest and relaxation [16]. Typical SpO_2_ values for healthy adults range from 95% to 99%. A higher SpO_2_ indicates a better systemic oxygen delivery that allows for better general performance [17].

#### 2.3.3. Data Analysis

NBI programs were designed as a pre–post test. The results were based on demographic data and performed measurements. Quantitative analyses were conducted with Statistica 13 software (StatSoft, TIBCO Software Inc., Palo Alto, CA, USA). A paired sample t-test was used to compare the difference between the pre-test and post-test data, and *p* < 0.05 was considered significant.

### 2.4. Ethical Considerations

The Committee for Research Ethics with the Participation of People of the University of Agriculture in Kraków provided ethical clearance for this study (protocol code 116/2023; date of approval 16 May 2023). The purpose and procedures of the study were explained to potential subjects by research staff. All of the participants were recruited on a voluntary basis and were guaranteed anonymity. A written informed consent was obtained from each individual. The participants were also assured that they had full rights to withdraw from the study at any time without any adverse consequence. All sessions were conducted by a qualified therapist who ensured that the best interest of the participants was guaranteed.

## 3. Results

### 3.1. Psychological Parameters

We observed positive changes in seniors’ self-esteem (SES) and well-being (WHO-5) measured at the baseline (that is at the beginning of the NBI programs) and at the end of four NBI programs, as confirmed by statistical analyses (Table 5). The mean score for all respondents after completing the NBI programs increased by on average 3 points for SES and 4 points for WHO-5. Larger growth, that is, greater improvement in self-esteem and well-being, was observed for the passive NBI programs (3.3 and 5.1 points, respectively) than in the active ones (2.6 and 3, respectively).

Detailed analyses confirmed the positive impact of the NBI programs on the tested parameters. After completing the NBI programs, we showed a higher score (as measured by SES and WHO-5) for both active and passive programs among the students of the U3A and the LTCF residents (Table 6). The resulting SES point increase ranged from 2.1 to 3.5. In both groups, better effects were observed following participation in the passive NBI programs. The greatest improvement in self-esteem was found in the LTCF residents participating in the passive NBI programs, and the smallest among the U3A students taking part in the active programs.

For well-being (WHO-5), better results were obtained in the U3A students completing the active programs, and in the LTCF residents, the passive programs seemed more effective. The obtained WHO-5 results showed an increasing trend ranging from 2.2 to 6.7. The greatest improvement (6.7) in well-being was found in the LTCF residents participating in the passive NBI programs, and the smallest (2.2), in the same group taking part in the active programs.

### 3.2. Physiological Parameters

Baseline PR and blood SpO_2_ measured before each 1.5 h session were at a similar level in the participants of the active and passive NBI programs, and the average of 76.7 bpm for PR and 96% for SpO_2_ fell within the normal limits for adults (Table 7). We demonstrated positive effects of all the sessions conducted during the NBI programs, which manifested in a lowered PR and increased SpO_2_ at the end of each session. For PR, the greater differences (decrease) before vs. after the sessions indicates the greater efficiency of the offered activities. We observed a greater decrease in PR for the sessions included in the passive programs (2.8 points) compared to the active programs (2 points). For SpO_2_, a greater increase was measured after participation in the passive NBI programs (1.2 points) than the active ones (0.7 points).

**Figure 2:**
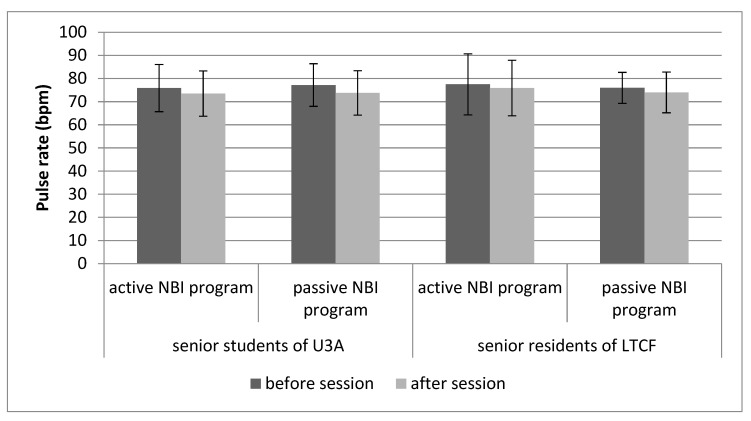
Effects of sessions of the nature-based intervention (NBI) programs on the pulse rate (PR) of University of the Third Age (U3A) students and residents of the long-term care facility (LTCF) measured before and after each session.

**Figure 3:**
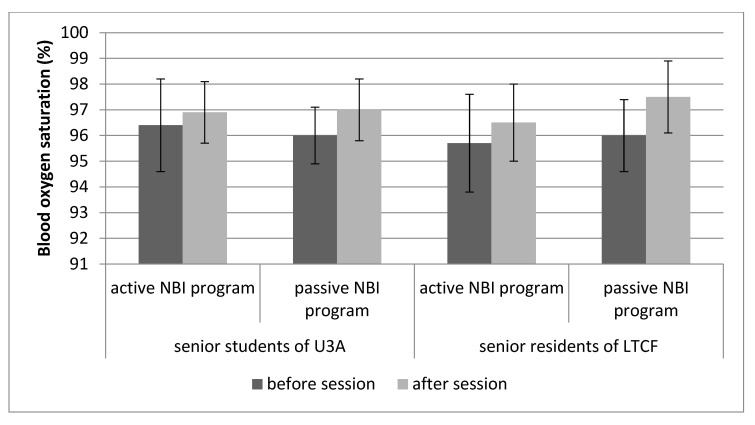
Effects of sessions of the nature-based intervention (NBI) programs on blood oxygen saturation (SpO_2_) of University of the Third Age (U3A) students and residents of the long-term care facility (LTCF) measured before and after each session.

The results of the paired samples t-test indicated significant differences in PR and SpO_2_ values following participation in the NBI program sessions (Figure 2 and Figure 3). The greatest (3.4 points) decrease in PR, as measured before and after the session, was found among the U3A students participating in the passive NBI programs. This score was approximately 1.5-times greater than that obtained among the LTCF residents participating in the passive NBI programs. For the active NBI programs, a greater decrease in PR was found among the U3A students, and it was also 1.5-times greater than among the LTCF residents. We observed an SpO_2_ increase, ranging from 0.5% to 1.5%, following participation in all sessions of the NPI programs. The rise was more pronounced among the participants of the passive programs, both in the U3A students (1%) and the LTCF residents (1.5%). The smallest change was noted among the U3A students taking part in the active NBI program sessions.

## 4. Discussion

The first studies on the effects of nature on human health and well-being were published in the early 1980s. They involved the intentional use of natural environment, plants, and gardening to improve the physiological, psychological, cognitive, and social parameters of participants. The studies differed greatly in their methodology and the participant population, but they all demonstrated the positive effect of nature and plants on the mental and physical health of people [18,19,20].

The use of gardening and horticultural therapy as a means of maintaining or improving the health and well-being of older adults is gaining attention in international literature [21]. Importantly, older adults form a specific and diverse social group with different needs and abilities, both physical and mental. Studies on the elderly usually focus on LTCF residents and less frequently on older adults living independently [22,23]. Moreover, the participants are often characterized by specific conditions, e.g., patients with schizophrenia [24], dementia [25], or depression [26]. Our research is the first to compare the impact of horticultural therapy programs on LTCF residents and older adults living independently, to check whether their different lifestyles and health status significantly affect the psychological and physiological effects of participation in these programs.

Gardening is a familiar activity and passion of many seniors who enjoy spending their time in the garden and participating in gardening therapy classes. This form of therapy allows also for the easy adaptation of the activities to the needs and capabilities of the participants [10,21]. The gardening therapy may include active and passive activities. During the passive classes, participants are most often encouraged to be consciously present in the garden by enjoying a garden view, meditation and senses engagement, or talking about past gardening experiences. The active classes rather focus on performing certain activities, such as sowing, planting, or propagating plants, arranging bouquets, or making plant-based artistic designs [27]. Earlier studies compared the effects of therapeutic activities based on measurements performed before and after the program or sessions or measurements carried out in the study and control group. As the literature lacks publications comparing the effects of active and passive programs, we performed such an analysis [23]. In this study, we compared the psychological and physiological responses to active and passive nature-based intervention programs in two groups of seniors: LTCF residents and older adults living independently.

Psychological tests assessing the effects of the garden therapy are usually performed at the beginning and end of a series of classes. The classes may be of various duration, and in our case, there were eight sessions lasting 1.5 h each. The basic test used in self-esteem assessment is the Rosenberg’s Self-Esteem Scale (SES). Self-esteem is important for a happy and fulfilling life and is a central aspect of psychological well-being [28]. However, research suggests that self-esteem decreases with age due to negative changes in social relationships, socioeconomic status, cognitive abilities, and health, being its crucial components [29]. This was confirmed in our study, where the average SES score was at baseline 40% lower than the average for the general population of Poland [11]. At the same time, the average score for older adults living independently was slightly higher than for LTCF residents.

Different therapeutic methods can be used to improve self-esteem. Studies show that one effective method can be gardening, which results in significant improvements in self-esteem and mood by reducing tension, depression, anger, and confusion [30]. Another method is exposure to nature through green exercise. Research shows that short-duration physical activity in green spaces provides a dose of nature that results in immediate mental health benefits, especially in people with a sedentary lifestyle, who are nonactive or mentally unwell [31]. Also, mindfulness has proven to have positive effects on self-esteem, which may be due to increased self-compassion, positive emotions, and acceptance achieved during mindfulness-based interventions [32]. This was confirmed in our research, which showed an increase in self-esteem following the participation of older adults in all nature-based intervention programs. We concluded that the passive programs brought about better results among both the LTCF residents and the older adults living independently than the active programs, which suggests that combining nature exposure with mindfulness is more effective.

Well-being can be assessed with various psychological tests, such as the World Health Organization Well-Being Index (WHO-5), Personal Well-Being Index (PWBI), General Well-Being Scale (GWB), or Ryff’s Scales of Psychological Well-Being (SPWB) [22,33]. In our study, the WHO-5 test confirmed that participation in the plant-related activities positively affected the well-being of the elderly. The effects of horticultural therapy on the well-being of older adults have also been studied by Perkins (2012), Lai et al. (2018), and Ng et al. (2018) [34,35,36], and all of them confirmed its beneficial impact on the well-being of the participants. However, it should be emphasized that the duration and type of classes, analyzed populations, and the assessment tests used in these studies varied considerably. The programs consisted of 6, 8, or 15 sessions lasting for 60 or 90 min. The offered activities included indoor and outdoor gardening, preparing plant-based daily necessities, cooking, and guided walks. The studies were conducted among residents of independent, community-based, age-restricted facility, nursing home resident, and independently living older adults. Well-being was assessed with the WHO-5, PWBI, and SPWB tests, respectively.

In our study, the WHO-5 test showed a greater improvement in well-being in the LTCF residents taking part in the passive programs, while a higher increase in well-being in older adults living independently was observed following participation in the active programs. With age, people change their perception of the importance of life domains, including their own well-being. For older adults living independently, important aspects of life that affect their well-being are maintaining a positive state of mind, engaging in worthwhile activities, and treating other persons properly [37]. Therefore, taking part in group art and craft activities aimed at creating specific items, helping each other, being valued, and focusing on the present improves their well-being. Also, introducing plants into such classes may trigger memories of the past in older people. Touching a specific plant or a scent of its flower may transport a person back to their childhood, which may enrich feelings of well-being for older adults [38,39]. On the other hand, for elderly people who need 24 h care, the important domains of life include strong mental energy, social connectedness, a sufficient number of meaningful activities, and a sense that their life has been worthwhile and that they have a meaningful future [37]. As a consequence, taking advantage of the restorative properties of nature by being outdoors may positively affect the well-being of these individuals [39]. This confirms the importance of creating and maintaining gardens at permanent residences for the elderly, to use them during passive classes. Moreover, the incorporation of breathing techniques, sensory stimulation, and mindfulness may also explain some of the observed benefits. An extensive body of research supports the claim that mindfulness may positively influence the well-being of older adults, as it is associated with a decline in loneliness, depression, and stress and an increase in general mood and positive emotions [40]. Sensory stimulation has also been shown to promote emotional, social, and occupational well-being, although the studies were conducted only among older adults with dementia [41].

In addition to the psychological tests used in research on the effects of therapeutic activities, physiological measurements, such as pulse rate or blood oxygen saturation, which we used in our study before and after each 1.5 h session, are important metrics. PR and SpO_2_ were used to evaluate the state of relaxation, which is an element of well-being. Previous studies have indicated that during relaxation, PR decreases and breathing becomes slower and deeper [9]. All nature-based intervention programs implemented in our study resulted in a decrease in PR and an increase in SpO_2_. Lowering PR through passive and active horticultural therapy was previously described by, for example, Goto et al. [42], whose study involved the viewing of a Japanese garden by people with a cognitive impairment; Zhao et al. [43], who reported peony viewing by adults; and Tu et al. [9], who implemented horticultural activities among middle-aged and elderly individuals. Zhao et al. [43] showed there was no effect of peony viewing on SpO_2_ levels, and Zeng et al. [44] demonstrated an increase in SpO_2_ levels among students following a bamboo forest therapy. Our study found that the passive programs brought about better relaxation effects than the active ones, which confirms that performing activities that result in a material effect (active therapy) has a smaller impact on the level of relaxation than consciously enjoying a garden (passive therapy). Similar conclusions were drawn by Lu et al. [45] based on a meta-analysis which showed that activities stimulating the five senses, such as walking, meditation, and tasting or smelling are more effective in stress reduction than handcrafting, flower arrangement, and gardening.

## 5. Conclusions

Our study contributes to the body of literature by showing the impact of horticultural therapy classes on improving the self-esteem, well-being, and relaxation of older people of different ages and living situations. Our research showed that horticultural therapy, as a form of nature-based intervention, plays an important role in improving self-esteem, well-being, and the state of relaxation in the elderly. By examining passive and active nature-based interventions in two different groups of U3A students and LTCF residents, we showed the beneficial effects of individual sessions and the entire programs on improving the self-esteem, well-being, and physiological relaxation of the participants. After each NBI session, we noted a decrease in PR and an increase in blood oxygen saturation, which indicated a deeper state of relaxation. In both groups, the self-esteem and well-being improved following the 8-week program, irrespective of the type of intervention.

Our study indicated also the need to carefully identify the group that is the target of the therapy. While active gardening programs offer substantial benefits, they are particularly effective in independent seniors, as shown by our group of U3A students. We also confirmed the fundamental role of creating green spaces in the residential areas of the elderly and around nursing homes. Our study indicated a greater improvement in the investigated parameters as a result of the passive NBI programs. Green areas are essential to improving the quality of life and promoting well-being in this demographic group.

## Figures and Tables

**Table 1:** Characteristics of the participants of the nature-based intervention programs—senior students of the University of the Third Age (U3A) and senior residents of the long-term care facility (UTCF).

Category	Participants			
	Students of the University of the Third Age (U3A)		Residents of the Long-Term Care Facility (LTCF)	
	*n*	%	*n*	%
Gender				
Female	22	91.7	21	87.5
Male	2	8.3	3	12.5
Age (years)				
60–70	12	50.0	6	25.0
71–80	12	50.0	4	16.7
>81	0	0	14	58.3

**Table 2:** Session schedule of the nature-based intervention program.

Nature-Based Intervention Program		
Session	Active	Passive
1	Sowing	Sensory walk—sight
2	Plant propagation	Sensory walk—hearing
3	Dry flower art	Sensory walk—smell
4	Pot painting	Sensory walk—touch
5	Paper succulent making	Sensory walk—taste
6	Bouquet making	Sensory walk—five senses walk
7	Flower arrangement in a basket	Mindfulness walk—gratitude
8	Wreath making	Mindfulness walk—happiness

**Table 3:** Exemplary session of the active nature-based intervention program.

Session: 2	Topic: Plant Propagation	Duration: 1.5 h
Aim of the session: engaging the participants, improving their well-being, expanding their interests, manual dexterity training, memory training, memory stimulation		
Course of the session		
5 min	Pre-session measurements	Pulse rate and blood oxygen saturation
5 min	Welcome and introduction to the session	Individual welcome to the participants, including questions about their well-being, the course of the day, and the condition of the plants from the previous session Short presentation of plant propagation and materials used during the session
10 min	Activity explanation	Presentation of plants used during the session Presentation of the various methods of vegetative plant reproduction
60 min	Plant-related activities	Making cuttings from shoots, leaves, and stolons Discussion about growing conditions and the basics of caring for plants used during the session Initiating conversations about pot plants Evoking memories from youth related to plants grown at home
5 min	Closing of the session	Session summary Time to share reflections
5 min	Post-session measurements	Pulse rate and blood oxygen saturation

**Table 4:** Exemplary session of the passive nature-based intervention program.

Session: 1	Topic: Sensory Walk—Sight	Duration: 1.5 h
Aim of the session: rest and relaxation, well-being improvement, perception improvement, establishing and maintaining social bonds, sense simulation, nature enjoyment		
Course of the session		
5 min	Pre-session measurements	Pulse rate and blood oxygen saturation
5 min	Welcome and introduction to the session	Individual welcome to the participants, including questions about their well-being and the course of the day Short presentation of the exercises and materials used during the session
10 min	Breathing exercises	Mouth breathing: “3-3-6 breathing technique”
60 min	Sensory activities and time to be in the garden	Focusing the eyes on the part of the landscape between the thumbs and widening the field of vision Zooming in and out of a selected natural object using a paper frame Seeing colors and patterns in nature (color wheel) Observation of nature Conversation and sharing experiences and thoughts
5 min	Closing of the session	Session summary Time to share reflections
5 min	Post-session measurements	Pulse rate and blood oxygen saturation

**Table 5:** Effects of the nature-based intervention (NBI) programs on the self-esteem (SES) and well-being (WHO-5) of the participants.

	Self-Esteem (SES)			Well-Being (WHO-5)		
	Baseline	End of the Program	Paired *t*-Test Statistics	Baseline	End of the Program	Paired *t*-Test Statistics
All participants	18.2 (4.2) *	21.2 (4.4)	t = 8.11 *p* = 0.001	15.7 (4.4)	19.7 (3.4)	t = 7.85 *p* = 0.001
Active NBI programs	18.7 (4.8)	21.3 (5.0)	t = 4.22 *p* = 0.001	16.5 (4.8)	19.5 (4.3)	t = 4.74 *p* = 0.001
Passive NBI programs	17.8 (3.5)	21.1 (3.7)	t = 8.59 *p* = 0.001	14.9 (4.0)	20.0 (2.2)	t = 6.59 *p* = 0.001

Mean ( ) *—standard deviation (SD).

**Table 6:** Effects of the nature-based intervention (NBI) programs on the self-esteem (SES) and well-being (WHO-5) of students of the University of the Third Age (U3A) and residents of the long-term care facility (LTCF).

	Nature-Based Intervention Program					
	Active			Passive		
	Baseline	End of the Program	Paired *t*-Test Statistics	Baseline	End of the Program	Paired *t*-Test Statistics
Self-esteem (SES)						
U3A students	19.8 (5.5) *	21.9 (6.0)	t = 2.82 *p* = 0.017	17.3 (2.7)	20.4 (3.3)	t = 6.82 *p* = 0.001
LTCF residents	17.6 (4.0)	20.7 (4.0)	t = 3.09 *p* = 0.01	18.3 (4.3)	21.8 (4.2)	t = 5.52 *p* = 0.001
Well-being (WHO-5)						
U3A students	14.8 (3.4)	18.6 (4.3)	t = 4.28 *p* = 0.001	15.9 (3.4)	19.4 (2.3)	t = 4.36 *p* = 0.001
LTCF residents	18.2 (5.5)	20.4 (5.5)	t = 2.5 *p* = 0.029	13.8 (4.4)	20.5 (2.0)	t = 5.67 *p* = 0.001

Mean ( ) *—standard deviation (SD).

**Table 7:** Effects of the nature-based intervention (NBI) programs on participants’ pulse rate (PR) and blood oxygen saturation (SpO_2_) measured before and after each session.

	Pulse Rate (PR) (bpm)			Blood Oxygen Saturation (SpO_2_) (%)		
	Before Session	After Session	Paired *t*-Test Statistics	Before Session	After Session	Paired *t*-Test Statistics
All participants	76.7 (10.1) *	74.3 (10.2)	t = 6.29 *p* = 0.001	96.0 (1.6)	97.0 (1.4)	t = 11.3 *p* = 0.001
Active NBI programs	76.7 (11.7)	74.7 (11.0)	t = 3.23 *p* = 0.001	96.0 (1.8)	96.7 (1.4)	t = 4.61 *p* = 0.001
Passive NBI programs	76.7 (8.1)	73.9 (9.2)	t = 6.76 *p* = 0.001	96.0 (1.3)	97.2 (1.3)	t = 14.68 *p* = 0.001

Mean ( ) *—standard deviation (SD).

## Data Availability

The raw data supporting the conclusions of this article will be made available by the authors on request.
